# Determination of the minimum soil infiltration rate of sunken green space considering the annual runoff collection ratio, sunken depth and sunken green space area of Hefei city, China

**DOI:** 10.1371/journal.pone.0299630

**Published:** 2024-03-05

**Authors:** Peigui Liu, Shuoya Cheng, Manting Shang, Zongsheng Wang, Song Wei

**Affiliations:** School of Civil Engineering, Hefei University of Technology, Hefei, 230009, China; Digital University Kerala, INDIA

## Abstract

Sunken green space is one of the urban rainwater collection facilities, which belongs to Low Impact Development (LID) techniques. It plays a key role in the construction of sponge city, and the amount of runoff collection is usually affected by the area of the sunken green space, the infiltration rate of the soil, and the annual runoff collection rate. To determine the minimum soil infiltration rate of sunken green space considering the annual runoff collection ratio of sponge cities, this paper selects a residential district in Hefei city, China, as the case study. Based on 45 years of precipitation data, the designed rainfall corresponding to annual runoff collection ratios of 75%, 80% and 85% is 21.3 mm, 23.4 mm and 27.5 mm, respectively. The characteristics of rainfall infiltration in sunken green space are analyzed by using the water balance model and runoff yield and conflux model. The results reveal that the soil infiltration rate is 1.16×10^−4^ cm/s~3.88×10^−3^ cm/s when the sunken depth is 0.1 m~0.3 m and that the ratio of green space area is 5%~25%. The runoff collection of the reconstructed sunken green space is 2.87 times and 1.98 times that of the nonsunken green space and the nonreconstructed sunken green space, respectively. That is to say, under the comprehensive performance of the sunken depth, sunken green space area, the steady soil infiltration rater of the reconstructed sunken green space cannot be less than the value obtained in this paper. Otherwise, the requirements of annual total runoff reduction ratio of the sponge city cannot be met. Therefore, this study provides references for realizing the ratio of annual runoff collection and sponge city construction in similar urban areas. It can also be applied to optimal selection of sunken green space in some sponge city projects.

## Introduction

With urbanization, the underlying urban surface conditions have dramatically changed, and the proportion of impervious areas has increased, which has reduced the rainfall infiltration ratio and increased surface runoff. Urbanization also affects the hydrological cycle under natural conditions [1–3]. Burns *et al*. [4] investigated the rainfall data of 27 sites in three representative basins in a highly urbanized area, a moderately urbanized area, and an undeveloped area of New York, USA, and found that peak flood discharge increased with the level of urbanization. White and Greer [5] explored the hydrological effect in California, USA, and discovered that the dry season discharge and flood peak discharge showed a significant increasing trend with the intensification of urbanization. Nie *et al*. [6] used the Soil and Water Assessment Tool (SWAT) to quantitatively analyze land use change in the upper San Pedro River Basin. Their results revealed that urbanization is the key factor of surface runoff increase, infiltration decrease and increased concentration of pollutants. Rapid urbanization causes not only serious flooding and ecological damage [7,8] but also declining groundwater levels in urban areas [9,10]. Therefore, efficient collection and utilization of rainfall have a role in reducing peak flood flow, reducing the municipal pipeline load and reducing urban nonpoint source pollution.

The annual runoff collection rate is a key control index in urban rainwater collection, utilization, and construction. The annual runoff collection rate refers to the proportion of accumulated annual collected runoff in the total annual runoff in a study area by natural and artificial measures such as infiltration, accumulation, utilization, evaporation and transpiration [11–13]. As the main permeable land surface of a city, urban green space is a natural green rainwater infrastructure that has a good rainfall runoff storage effect [14–16], has a significant role in controlling the total amount of runoff, and has a low construction cost. By monitoring the operation of green spaces during 28 rainfall events at Monash University in Australia, Hatt *et al*. [17] concluded that the average flood peak reduction rate of green space reached 80%. Sunken green space is a kind of public green space whose elevation is lower than the surrounding road surface, and the plants in the green space are mainly herbaceous. Compared with bioretention, bioswale, or vegetated swale, it has the characteristics of large area, short and small plants. It is usually built on both sides of the road. The typical structural characteristic of sunken green space is that its ground elevation is lower than that of the surrounding ground, so runoff from the surrounding area naturally flows into the sunken green space under the action of gravity. Yang *et al*. [18] concluded that the rainfall infiltration and storage effect of sunken green space are more effective than those of flat green space. The main factors of the rainfall infiltration and storage capacity of sunken green space are the rainfall intensity, sunken depth of green space, sunken green space area and steady infiltration rate of soil [19].

Japan initiated a rainfall infiltration and storage plan to replenish groundwater and to improve the ecological environment by transforming public land into infiltration sites in 1980 and proposed measures such as green space maintenance, reservoir regulation, rainwater storage and infiltration promotion to achieve a healthy water cycle [20]. New Zealand enacted the Resource Management Act in 1991, which sets out specific requirements for controlling surface runoff by regional governments, including the application of stormwater runoff source control measures, and the construction of centralized regulation facilities to regulate delayed stormwater discharge. The United States Environmental Protection Agency promoted the concept of green infrastructure in 2007, which includes large-scale facilities that are designed to effectively control heavy rain events and to protect ecosystems [21,22]. Traditional infrastructure and green infrastructure are closely related. Australia proposed the Water Sensitive Urban Design (WSUD) in response to the country’s urban characteristics. The core approach of the WSUD is to use the urban water cycle; consider the management of rainwater, water supply and total reclaimed water; advocate the combination of hydrological design and urban planning; and minimize the impact of urban development on the water cycle. Kuller *et al*. [23] have shown that the annual runoff collection ratio of a new urban area and low-impact development rainwater systems can reach 80%~85% by controlling the high frequency of small and medium-sized rainfall events.

Sunken green space is a type of urban green infrastructure that has significant ecological benefits in improving urban vegetation coverage, enhancing urban ecological environment, alleviating urban heat island effect, and promoting rainwater utilization. Currently, terrain analysis, ecological simulation, hydrological simulation, water quality monitoring, and remote sensing technology are gradually being applied to the study and management of sunken green space. These methods can effectively evaluate the ecological function and social value of sunken green space, providing a scientific basis for its design and management. At present, most of the research topics of the sunken green space have focused on how to improve the rainwater collection, and the hydrological process or characteristics of the green space. The research methods include experimental and numerical model. However, there are still some problems in practical design and management, such as the structure and storage capacity of sunken green space not matching, and the use of soil infiltration coefficients being either too small or too large. Sunken green space is one of the low-impact development measures of sponge city. The construction goal of sponge city is to improve the annual total runoff collection ratio, which reflects the annual runoff reduction. The reduced runoff is temporarily stored under the surface through infiltration. Therefore, the infiltration rate of low-impact development measures is the main factor affecting the annual runoff reduction. If this parameter is ignored during the design of sunken green space, it is easy to cause the mismatch between the designed sunken green space structure and the rainwater collection ratio. It may result in reduction of rainwater collection amount or increase the total construction cost of sunken green space. In order to meet the requirements of annual runoff collection rate in sponge city, it is necessary to calculate the soil infiltration rate scientifically to promote the development and improvement of sunken green space.

The increase in impervious areas via high-density urbanization means that there is not enough natural green space to effectively store rainwater [24,25]. Presently, there are two main problems in urban green space construction. The first problem is that the function of urban green space is mainly to beautify the environment and mitigate the lack of a rainwater runoff regulation function. Second, to avoid flooding plant roots, most of the green space structure is mainly flat, which is not conducive to rainwater infiltration. To increase the infiltration of surface runoff in urbanized areas, we selected a residential area in Hefei City, China, as the case study. Considering the total annual runoff control rate of rainwater, the minimum soil infiltration rate of sunken green space is calculated. The influence of sunken green space on surface runoff is analyzed by using the water balance model, which provides references for realizing the ratio of annual runoff collection and sponge city construction in urban areas.

## Methodology

### Water balance model of sunken green space

**Fig 1:**
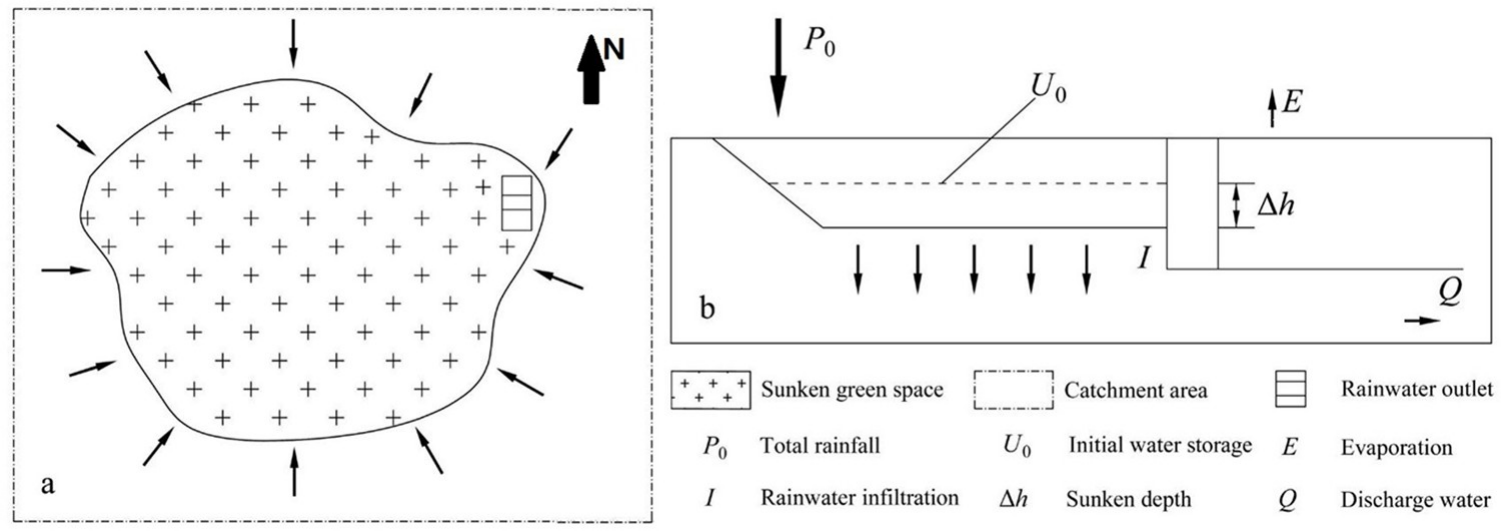
Schematic diagram of sunken green space.

It is assumed that all the surface runoff in the catchment area flows into the sunken green space during rainfall. When the inflow exceeds the storage capacity of the sunken green space, rainwater is discharged through the overflow outlet set in the green space (Fig 1).

According to the principle of water balance, the relationship between inflow and outflow is expressed as follows:

$$P_{0}+U_{0}=D+E+I+U_{1}+Q$$ (1)

where *P*_0_ is the total volume of one rainfall event, in m^3^; *U*_0_ is the initial water storage of the green space, in m^3^; *D* is the surface runoff loss in one rainfall event, in m^3^; *E* is evaporation during rainfall, in m^3^; *I* is the rainfall infiltration, in m^3^; *U*_1_ is the water storage of the green space at the end of rainfall, in m^3^; and *Q* is the water discharged through the green space, in m^3^.

The calculation period of Eq (1) is generally the duration of a rainfall event. In this period, evaporation comprises a very small proportion in relation to the total rainfall, which is zero at this time. It is assumed that there is no water storage in the sunken green space when the rainfall starts, that is, *U*_*0*_ = 0. To improve the utilization rate of precipitation, all surface runoff is set to be stored in the sunken green space without outflow, that is, *Q =* 0. Then, the water balance Eq (1) is simplified as:

$$P_{0}=D+I+U_{1}$$ (2)

### Runoff calculation

The catchment area of sunken green space *S* is usually composed of two parts, a pervious area and an impervious area, with pits for water storage in impervious areas. The runoff-producing area *S* of the sunken green space catchment is divided into four subregions: impervious area *S*_*1*_ without pit water storage, impervious area *S*_*2*_ with pit water storage, permeable area *S*_*3*_ without sunken green space, and sunken green space *S*_*4*_. The relation expression is *S* = *S*_*1*_+*S*_*2*_+*S*_*3*_+*S*_*4*_.

The runoff yield of impervious areas without pit water storage is:

$$W_{1}=P_{z}\cdot S_{1}\cdot C_{1}$$ (3)

where *W*_*1*_ is the runoff yield in the impermeable area in precipitation duration, in m^3^; *P*_*z*_ is the rainfall, in mm; and *C*_*1*_ is the coefficient of surface runoff in the impervious area.

The runoff yield of the impervious area with pit water storage is:

$$W_{2}=P_{z}\cdot S_{2}\cdot C_{1}-Q_{x}$$ (4)

where *W*_*2*_ is the runoff yield in the impermeable area with pit water storage in precipitation duration, in m^3^; *Q*_*x*_ is the water storage of the pit, in m^3^.

The runoff yield of permeable areas without sunken green space is:

$$W_{3}=P_{z}\cdot S_{3}\cdot C_{2}$$ (5)

where *W*_*3*_ is the runoff yield in the permeable area without sunken green space in precipitation duration, in m^3^; *C*_*2*_ is the coefficient of surface runoff in the impervious area.

The runoff yield in the sunken green space is:

$$W_{4}=P_{z}\cdot S_{4}$$ (6)

where *W*_*4*_ is the runoff yield in the sunken green area in precipitation duration, in m^3^.

It is assumed that all the runoff yield in the catchment area is collected to the sunken green space. According to Eqs (3)–(6), the runoff yield *W* in the catchment area is:

$$W=W_{1}+W_{2}+W_{3}+W_{4}$$ (7)

### Determination of the minimum soil infiltration rate of sunken green space

The main factors that determine the rainfall collection effect of sunken green space are the soil infiltration rate, sunken depth and the area of sunken green space and so on [25]. As for the factor of soil infiltration rate, if the value is too large, it may cause an increase in the construction cost of water storage space and affect the normal growth of vegetation in sunken green space. If the value is too small, it may lead to a reduction in the rainwater collection ability, a short delay time of the flood peak, and a failure to achieve the total annual runoff collection ratio. Therefore, it is necessary to scientifically calculate the soil infiltration rate of sunken green space. And the soil infiltration rate is the most key index to determine the amount of rainwater infiltration through the sunken green space. If we ignore the soil infiltration rate and only increase the area of the sunken green space, the construction cost will increase, and the land use structure may be unreasonable [25].

The rainfall collection rate of sunken green space refers to the ratio of the sum of rainwater infiltration and rainwater storage to the total rainwater runoff in the catchment area of the sunken green space, which is expressed as Eq (8). When all runoff is infiltrated by sunken green space and the outflow is zero, the ratio is 100%.

$$N=\frac{I+U_{1}}{W}\times 100\%$$ (8)

$$I=KJS_{4}t$$ (9)

where *N* is the rainwater collection rate of the sunken green space; *K* is the soil infiltration rate of the sunken green space, in cm/s; *J* is the hydraulic slope; and *t* is the infiltration time, in minutes.

The structure of sunken green space is generalized as trapezoidal. The equation for calculating its water storage is expressed as:

$$U_{1}=\frac{(S_{4}+{S_{4}}^{\prime })\Delta h}{2}$$ (10)

where Δ*h* is the sunken depth, namely, the difference in the water level between two states of storage within the sunken green space, in m, and *S*_4_′ is the bottom area of the sunken green space.

Substituting Eqs (3)–(6) and (9)–(10) into Eq (8) yields the following expression:

$$\begin{array}{l} N=\frac{K\cdot J\cdot S_{4}\cdot t+(S_{4}+{S_{4}}^{\prime })\Delta h/2}{P_{z}\cdot S_{1}\cdot C_{1}+(P_{z}\cdot S_{2}\cdot C_{1}-Q_{x})+P_{z}\cdot S_{3}\cdot C_{2}+P_{z}\cdot S_{4}}\times 100\%\\ \;=\frac{K\cdot J\cdot S_{4}\cdot t+(S_{4}+{S_{4}}^{\prime })\Delta h/2}{W}\times 100\% \end{array}$$ (11)

According to the theoretical analysis, to store much rainfall in the sunken green space, for a given infiltration area, the smaller the soil infiltration rate is, the greater the storage. However, the value cannot be too small because it cannot achieve the goal of the runoff collection rate. Since we assume that sunken green space is filled with rainwater at the end of a rainfall, the soil infiltration rate of the sunken green space is the smallest. There is vegetation in sunken green space, and the rainfall storage time should not exceed the waterlogging tolerance time of vegetation; otherwise, it will affect the normal growth of vegetation. According to the results of Ziegler *et al*. [26] and Zhang and Zhang [27], the limit of waterlogging tolerance time of sunken green space vegetation in Hefei is determined to be 72 h, that is, when the sunken green space reaches the maximum instantaneous storage after rainfall, the time required to be completely infiltrated by soil is 72 h. It is assumed that all runoff is infiltrated by sunken green space, that the outflow is zero, and that the ratio *N* is 100%. Then, the expression of the minimum soil infiltration rate is:

$$K_{\min}=\frac{\Delta hW-2(S_{4}+{S_{4}}^{\prime })}{\Delta hJS_{4}t}$$ (12)

### Data sources

This paper selects a residential area in Hefei City of Anhui province, China, as the case study. The data are derived from derived from the Hefei City Development Plan, Residential Community Construction Plans, and the Anhui Province Water Resources Bulletin.

## Case study

### Overview of the study area

This study selected a residential area in Hefei city, East China, as the research area. The area has a humid subtropical monsoon climate. The annual average temperature is 15.7°C, and the annual average rainfall is 1,000 mm. Rainfall mainly occurs from June to August, accounting for approximately 45% of annual rainfall. The soil is mainly composed of quaternary brown‒yellow clay and subclay, with heavy soil texture and heavy soil volume. Under natural conditions, the soil infiltration rate in the study area is approximately 5.85×10^−5^ cm/s [28].

The length of the east and west sides of the residential district is about 324m and 302m, and the length of the south and north sides is about 182m and 195m, respectively. The rainfall catchment area of the residential district is approximately 58410.60 m^2^. The areas of roads and buildings are 12908.70 m^2^ and 23183.21 m^2^ respectively, accounting for 22.10% and 39.69%, respectively, of the total area. The impervious area and green area are 36091.91 m^2^ and 22318.69 m^2^, respectively, accounting for 61.79% and 38.21%, respectively, of the total area. There are plans to transform part of the green space in the residential area into sunken green space. The vegetation types are mainly medium-sized, low tree shrubs and ground-cover plants such as privet aureus and boxwood aureus. The residential area is built with rainwater and sewage diversion drainage systems.

### Annual runoff collection ratio

**Fig 2:**
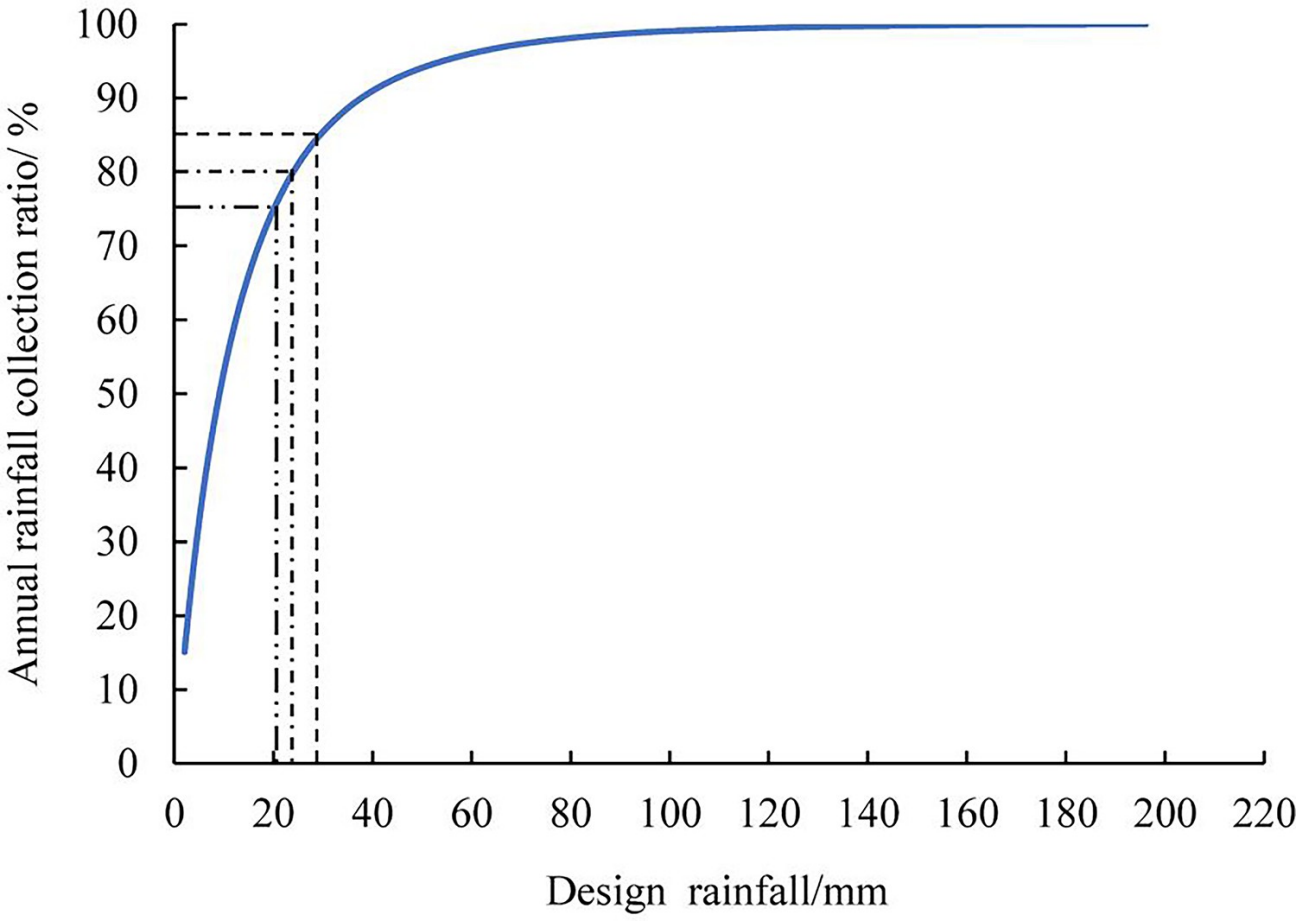
Designed rainfall.

The daily rainfall in Hefei city from 1975 to 2019 was analyzed. First, rainfall events less than or equal to 2 mm are deducted. Second, the proportion of the total rainfall less than a certain amount in the total rainfall is calculated by sorting the rainfall from small to large, which is referred to as the total annual runoff collection ratio *α*. According to The Technical Guide of Sponge City Construction in China, the total runoff collection ratio of Hefei city should fall between 75% and 85%. Therefore, this study uses the ratios as 75%, 80% and 85% for analysis. Based on the daily rainfall data from 1975 to 2019, the rainfall (also known as designed rainfall) corresponding to 75%, 80% and 85% annual runoff collection ratios was 21.3 mm, 23.4 mm and 27.5 mm, respectively (Fig 2).

## Results

### Determination of the minimum soil infiltration rate of sunken green space

The runoff coefficient *C*_1_ of the impervious area without depressions is 0.9, and the runoff coefficient *C*_2_ of the permeable area without sunken green space is 0.25. There is no impervious area with pits in the study area. Wu *et al*. [29] noted that when considering the infiltration capacity and the structural safety of green space, the depth of sunken green space should be fall in the range of 0.1 m-0.3 m. This is the actual situation in the Hefei area. Then, the sunken depth Δ*h* of the sunken green space is set to 0.1 m, 0.2 m, and 0.3 m. There are plans to transform part of the green space in the residential area into sunken green space. To analyze the relationship between the area ratio of sunken green space and the soil infiltration rate, the area ratio *f* is expressed as:

$$f=\frac{S_{4}}{S_{3}}\times 100\%$$ (13)

The area ratio *f* of sunken green space is set to 5%, 10%, 15%, 20% and 25% for calculation. The ratio refers to the proportion of sunken green space to the case study area. According to Eq (12), the relationship among sunken depth, area ratio of sunken green space, and the soil infiltration rate is obtained as shown in Table 1.

**Table 1:** Minimum soil infiltration rate with the annual runoff collection ratio.

Sunken depth *△h*	Annual runoff collection ratio *N/%*	Area ratio *f* of sunken green space/%				
		5	10	15	20	25
**0.1**	75	2.69×10^−3^	7.61×10^−4^	1.18×10^−4^	1.16×10^−4^	1.16×10^−4^
	80	3.09×10^−3^	9.73×10^−4^	2.67×10^−4^	1.16×10^−4^	1.16×10^−4^
	85	3.88×10^−3^	1.39×10^−3^	5.57×10^−4^	1.42×10^−4^	1.16×10^−4^
**0.2**	75	1.30×10^−3^	1.16×10^−4^	1.16×10^−4^	1.16×10^−4^	1.16×10^−4^
	80	1.70×10^−3^	1.16×10^−4^	1.16×10^−4^	1.16×10^−4^	1.16×10^−4^
	85	2.49×10^−3^	1.16×10^−4^	1.16×10^−4^	1.16×10^−4^	1.16×10^−4^
**0.3**	75	1.16×10^−4^	1.16×10^−4^	1.16×10^−4^	1.16×10^−4^	1.16×10^−4^
	80	3.13×10^−4^	1.16×10^−4^	1.16×10^−4^	1.16×10^−4^	1.16×10^−4^
	85	1.10×10^−3^	1.16×10^−4^	1.16×10^−4^	1.16×10^−4^	1.16×10^−4^

As listed in Table 1, for a given volume of rainwater to manage and a given design of the system, the necessary soil infiltration rate decreases as the area ratio of the sunken green space to the total catchment increases. If *N* = 75% and *△h* = 0.1 m, when the area ratio of sunken green space increases from 5% to 25%, the soil infiltration rate decreases from 2.69×10^−3^ cm/s to 1.16×10^−4^ cm/s. When *f* is constant, the necessary steady infiltration rate of soil K increases with increasing annual runoff collection ratio N. The necessary steady infiltration rate of soil decreases with increasing sunken depth. When *N* = 85%, *△h* = 0.1 m and *f* = 5%, the maximum soil infiltration rate of the sunken green space reaches 3.88×10^−3^ cm/s. When *N* = 75%, *△h* = 0.3 m, and the green space area ratio is 5%, the soil infiltration rate reaches the minimum value of 1.16×10^−4^ cm/s.

**Fig 3:**
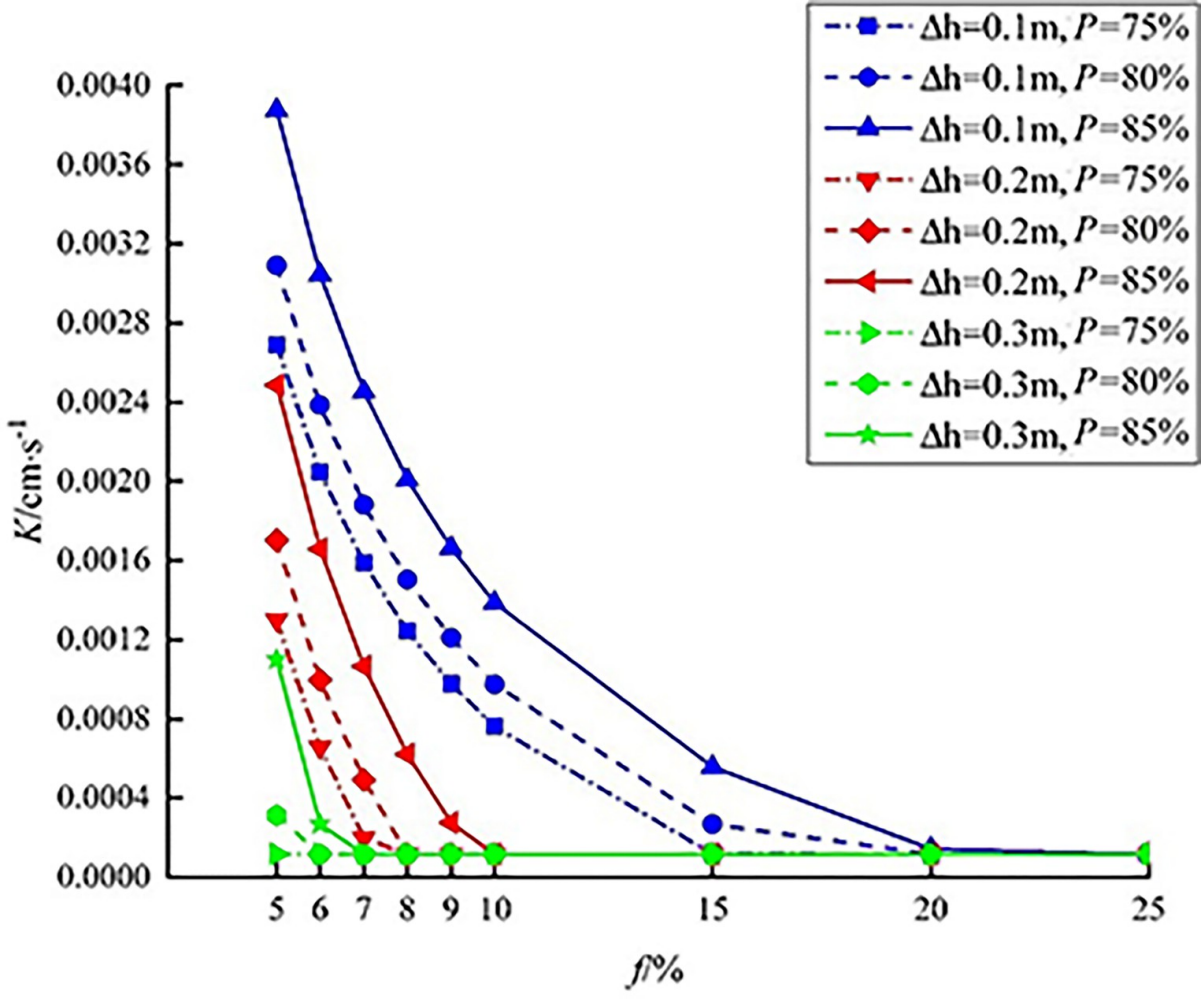
The minimum soil infiltration rate.

To further analyze the variation characteristics of the soil infiltration rate, the analysis results when *f* = 6%, 7%, 8% and 9% were added are shown in Fig 3. When the sunken depth is 0.1 m and the *f* value gradually increases to 15%, the *K* value decline rate is approximately 85%. When *f* increases from 15% to 20%, the decline rate drops to approximately 44%. When *f* continues to increase to 25%, the decline rate is approximately 5%, indicating that the soil infiltration rate hardly changes. When the sunken depth increases to 0.2 m, a *K* value decline rate greater than 85% is mainly reflected in the section where *f* is less than 10%, while when the sunken depth increases to 0.3 m, the *K* value decline rate is obvious in the section where *f* is less than 6%.

When the area ratio of sunken green space (≤5%) and the sunken depth (<0.1 m) are combined, the soil infiltration rate of sunken green space increases. For example, when the annual runoff control rate is 75%, *f* = 5%, and *△h* = 0.1 m, the soil infiltration rate of the sunken green space is 2.69×10^−3^ cm/s. However, when the higher sunken green space area ratio (≥15%) and the higher sunken depth (h>0.1 m) are combined, the soil infiltration rate of the sunken green space is relatively small, only 1.16×10^−4^ cm/s. In addition, the combination of a higher sunken greening rate (>25%) and lower sunken depth (<0.1 m) or the combination of a lower sunken rate (≤20%) and higher sunken depth (>0.2 m and 0.3 m) produces good results. Therefore, when *f* and *△h* are reasonably combined, the smaller soil infiltration rate of sunken green space achieves the target annual runoff collection rate.

### Analysis of rainfall collection capacity in sunken green space

Based on the water balance model, the differences in the rainfall collection capacity of nonsunken green space, untransformed green space and transformed sunken green space are analyzed when the sunken depth is 0.1 m and the designed rainfall is 21.3 mm, 23.4 mm and 27.5 mm, respectively. The results are shown in Table 2.

**Table 2:** Differences in rainfall collection capacity of nonsunken green space, untransformed sunken green space and transformed sunken green space.

Designed rainfall/mm	Total rainfall/m^3^	Rainfall collection/m^3^			Proportion of annually retained rainfall/%		
		Nonsunken green space	Untransformed sunken green space	Transformed sunken green space	Nonsunken green space	Untransformed sunken green space	Transformed sunken green space
**21.3**	1,244.15	433.42	627.05	1,244.15	34.8	50.4	100
**23.4**	1,366.81	475.65	688.87	1,366.81			
**27.5**	1,606.29	558.99	809.57	1,606.29			

As shown in Table 2, when no sunken green space is built in the study area, the amount of collected rainfall only accounts for 34.8% of the total rainfall. A total of 55.2% of runoff will be discharged to the rainwater pipe, which cannot be infiltrated; thus rainwater collection and utilization is not realized. When the existing soil infiltration capacity (5.85×10^−5^ cm/s) of the study area is used to construct the sunken green space, the proportion of rainfall collection reaches 50.4%. However, if the annual runoff collection ratio is set to 75%, the sunken green space needs to be reformed from 5.85×10^−5^ cm/s to 1.16×10^−4^ cm/s in Table 1. Then, the reconstructed sunken green space will retain nearly 100% of the annual rainfall, which is 2.87 times and 1.98 times that of the nonsunken green space and the nontransformed sunken green space, respectively. After the transformation, sunken green space has a significant effect on improving the capacity of rainfall infiltration and storage.

The rainfall retention efficiency of sunken green space may be related to the soil infiltration rate, sunken depth, sunken green space area ratio and other factors. Fig 3 shows that the soil infiltration rate decreases with increasing area ratio of sunken green space. When the sunken depth is 0.1 m and the sunken green space area ratio is greater than 20%, the soil infiltration rate is approximately 1.16×10^−4^ cm/s. When the sunken depth is 0.2 m and the sunken green space area ratio exceeds 10%, the soil infiltration rate is approximately 1.16×10^−4^ cm/s. When the sunken depth is 0.3 m and the sunken green space area ratio is greater than 7%, the soil infiltration rate is approximately 1.16×10^−4^ cm/s. To achieve the total annual runoff control goal, when the area ratio of the sunken green space is less than 20%, 10% and 7%, the soil infiltration rate should be increased. According to the research results of Wu *et al*. [29], green space with a sunken depth of 0.1 m has a wide range of applications. Therefore, using the sunken depth of 0.1 m in Fig 3 as an example, the mathematical relationship between the area ratio *f* and the soil infiltration rate *K* of sunken green space is obtained by the fitting method, as shown in Table 3.

**Table 3:** Relationship between the area ratio *f* of sunken green space and the soil infiltration rate *K* of sunken green space.

Sunken depth *△h*	Annual runoff collection ratio *N/%*	Mathematical expression	Coefficient of determination
**0.1**	75	$K=0.0069e^{-22.59f}$	0.9952
	80	$K=0.0089e^{-22.28f}$	0.9995
	85	$K=0.0121e^{-21.68f}$	0.9989

Table 3 shows that the relationship between the area ratio f and the soil infiltration rate K of sunken green space is an exponential curve. The coefficients of determination are greater than 0.99, indicating that the mathematical model fits well and can be used for prediction. Under the constraints of the sunken depth and annual runoff collection ratio, the mathematical expression in Table 3 can be used to calculate the soil infiltration rate that corresponds to any area ratio f of sunken green space and provides a technical basis for the design of rainfall collection measures.

## Discussion

The current study was an attempt to determine the infiltration coefficient of soil in the construction process of sunken green space, to achieve the annual runoff control rate requirements. A residential community in Hefei is taken as an example, and the analytical method is used to calculate the infiltration coefficient of soil. The results indicates that sunken green space can significantly increase stormwater storage. For the case study selected in this paper, when the undisturbed soil in the study area is used to construct sunken green space, the amount of rainwater runoff that is collected reaches 50.4%, which is 15.4% greater than that collected in traditional green space. According to the Technical Guidelines for Sponge City Construction in China, the minimum control rate of annual runoff collection in Hefei is 75%. Since 50.4% is less than 75%, the rainfall collection has not reached the target established in the guidelines for the study area. It is necessary to improve the soil permeability of sunken green spaces.

Sunken green spaces can be transformed to increase runoff collection. However, the operation of sunken green spaces may cause a blockage or decrease in the infiltration rate. This rate is not constant over time and is reduced, especially in sunken spaces, if real maintenance and monitoring measures are not implemented. How long can a sunken green space last? To answer this question, we need to carry out a sustainability evaluation of sunken green space, which will be the next step of our group’s in-depth study.

To improve the effect of rainfall collection, the minimum soil infiltration rate of sunken green space is calculated based on the theory of production and confluence and the water balance model for the residential district of Hefei city. The exponential function expression is obtained. The production and confluence theory and water balance model are applicable to other rainfall collection areas. Therefore, the calculation methods and ideas in this paper are applicable to other similar urban areas, and only the coefficients in the mathematical expressions are different.

## Conclusions

Green space has a very important role in beautifying the urban environment and purifying the air. Simultaneously, it is also one of the main measures for urban rainfall collection. However, traditional green space has a limited capacity for rainfall collection, and the effect of delayed flood peak or rainwater collection is not significant. Traditional green space needs to be rebuilt. Based on the annual runoff control rate issued by China, this paper analyzes the minimum soil infiltration rate in the reconstruction of common green space. The soil infiltration rate is 1.16×10^−4^ cm/s~3.88×10^−3^ cm/s when the corresponding depth of green space is 0.1 m~0.3 m and the ratio of green space area is 5%~25%. The value is related to the annual runoff collection ratio, sunken depth of green space and sunken green space area. When the annual runoff collection ratio and sunken depth are constant, the soil infiltration rate decreases with an increase in the area ratio of sunken green space. Especially when *f* <15%, the soil infiltration rate greatly decreases. When the area ratio of sunken green space to sunken depth is constant, the soil infiltration rate increases with increasing annual runoff control rate. The runoff collection amount of the reconstructed sunken green space is 2.87 times and 1.98 times that of the nonsunken green space and nonreconstructed sunken green space, respectively. The collection rate of annual runoff is achieved by the minimum soil infiltration rate in high-density urban areas when the sunken depth and area ratio of sunken green space are appropriately combined.

As one of the key measures for urban rainwater collection and utilization, sunken green space plays an important role in controlling peak flow and utilizing rainwater resources in the urban area. The soil infiltration coefficient of sunken green space directly determines the effectiveness of peak flow control and rainwater collection. Therefore, considering the constraints of sunken depth, the ratio of sunken green space area, and the annual runoff control rate, scientific calculation of the minimum soil infiltration rate can effectively enhance the function of sunken green space. In addition, the water balance model and runoff yield and conflux model were used to calculate the soil infiltration rate in this study. When the soil information and precipitation characteristics of some area are clear, the model can be applied to calculate the soil infiltration rate that meets the annual rainwater collection rate of sponge city. If the conditions of the study area are similar to those in this paper, the values obtained in this study can be used as reference for the design of the sunken green space. They may also be not applicable in case of a second rain event soon after the first. And it is more suitable for semi-humid, arid and semi-arid areas.

## Supporting information

S1 Data (XLS)
